# Assessment of patient information needs: A systematic review of measures

**DOI:** 10.1371/journal.pone.0209165

**Published:** 2019-01-31

**Authors:** Eva Christalle, Jördis M. Zill, Wiebke Frerichs, Martin Härter, Yvonne Nestoriuc, Jörg Dirmaier, Isabelle Scholl

**Affiliations:** 1 Department of Medical Psychology, University Medical Center Hamburg-Eppendorf, Hamburg, Germany; 2 Department of Psychosomatic Medicine and Psychotherapy, University Medical Center Hamburg-Eppendorf, Hamburg and Schön Clinic Hamburg Eilbek, Hamburg, Germany; Weill Cornell Medical College in Qatar, QATAR

## Abstract

**Background:**

Providing patient information is a central aspect of patient-centered care. Fulfilling personal information needs has positive effects on several health-related outcomes. Measurement instruments help to identify individual information needs in an effective way. The present study gives an overview of existing information needs measures and further evaluates the quality of their psychometric properties and their psychometric studies.

**Methods:**

We conducted a systematic search on psychometric studies of measures that assess information needs in PubMed and Embase. Furthermore, we carried out a secondary search with reference and citation tracking of the included articles. Title, abstracts and full texts were screened by two independent reviewers for eligibility. We extracted data on content of the measures, validation samples and psychometric properties. In addition we rated the methodological quality with the COSMIN checklist and the quality of psychometric properties with the criteria of Terwee and colleagues.

**Results:**

24 studies on 21 measures were included. Most instruments assessed information needs of patients with cancer or cardiac diseases. The majority of the instruments were in English language and from western countries. Most studies included information on internal consistency and content validity. The ratings showed mixed results with clear deficiencies in the methodological quality of most studies.

**Discussion:**

This is the first systematic review that summarized the existing evidence on measures on patient information needs using two instruments for a systematic quality assessment. The results show a need for more psychometric studies on existing measures. In addition, reporting on psychometric studies needs to be improved to be able to evaluate the reliability of the psychometric properties. Furthermore, we were not able to identify any measures on information needs for some frequent chronic diseases. Other methods to elicit information needs (e.g. open-ended interviews, question prompt sheets) could be considered as alternatives if sound measures are missing.

## Introduction

Patient information has been found to be one of the most important dimensions of patient-centered care [1, 2]. Meeting information needs increases treatment satisfaction [3] and aids informed decision making [4]. Furthermore, the provision of disease related information is an important determinant for patient-reported health-related outcomes (e.g. treatment adherence, emotional and psychological health/wellbeing, quality of life) [5–7].

Studies show that patients often do not receive satisfactory information from their healthcare provider (HCP). [5, 8]. Reasons for these findings are multiple. On the one hand, HCPs tend to underestimate patients’ information needs and may as well overestimate the amount of information given [5]. Misunderstandings between patient and clinician can lead to missing or false information and/or different appraisal or perception of the importance of information [5, 9]. In addition, some HCPs lack the skills to assess the patients’ information needs and to provide tailored information. Especially when facing a life-threatening disease, giving the right amount of information to the patient has been found challenging for the HCP as HCPs often assume that too much information might harm [5]. On the other hand, patients themselves have been found insecure or reluctant to voice their personal information needs [5, 10].

Nonetheless, providing all potentially relevant information can be problematic too. Too much information can lead to a cognitive overload for the patients. With this overload patients tend to forget relevant information or feel stressed. Not all patients wish for all information [11] and the exact information needs can vary individually [8, 12]. Thus, it is important to assess individual needs and tailor information accordingly [10]. Health communication tailored to individual needs enhances the information processed by the patient and supports motivation and behavior changes [13].

The very first step to satisfy individual information needs is to elicit and understand these [14]. Standardized measurement instruments aid to uncover those needs and allow to give relevant information to the patient. For this reason, psychometrically sound measures are needed to assess individual information needs.

Therefore we aimed to identify studies assessing psychometric properties of measures capturing patient information needs, to evaluate the methodological quality of these studies and to assess the quality of the psychometric properties of the identified measures. Overall, this is the first review which provides a comprehensive overview of existing information needs measures and their quality.

## Methods

The systematic review was registered in the PROSPERO International Prospective Register of Systematic Reviews (registration code: CRD42014012867). The PRISMA Checklist can be found in S1 Appendix.

### Search strategy

We performed an electronic literature search in the databases PubMed and EMBASE, including all articles from their inception to July 16, 2014 and ran an update with the same search strategy on May 31, 2016. The search strategy consisted of a combination of different terms and keywords from the following four domains: (i) construct, (ii) context, (iii) measurement, and (iv) psychometrics (see Table 1). The same search strategy had been used for both databases. No limitations had been applied. Our primary electronic search strategy was complemented by a secondary search, consisting of reference and citation tracking of included articles.

**Table 1:** Search strategy for electronic database search.

**Construct**	(information OR informational) AND (need OR needs OR requirement OR requirements OR want OR wants OR preference OR preferences)
**Context**	patient OR patients
**Measurement**	instrument OR instruments OR measurement OR measurements OR measure OR assessment OR assess OR tool OR tools OR questionnaire OR questionnaires
**Psychometrics**	validity OR reliability OR reliable OR "internal consistency" OR validation OR validate OR psychometric OR psychometrical OR "factor analysis"
**Final results**	**(Construct) AND (Context) AND (Measurement) AND (Psychometrics)**

### Eligibility criteria

We retrieved peer-reviewed publications, published in English or German. We included studies, which tested psychometric properties of measures that assess the construct patient information needs. Our definition was based on Timmins [8], who defined information needs as “personal expressed needs of the client […] for specific condition-related information. Information needs are therefore expressed needs, rather than normative (defined by the professional).” For the purpose of this review we included measures on expressed needs for treatment-related information as well. We focused on adult patients only and excluded measures that assessed information needs as a subscale of a broader measure. The applied inclusion and exclusion criteria are displayed in Table 2.

**Table 2:** Exclusion criteria.

**E1**	The full text is NOT available (neither via internet nor library).
**E2**	The language of publication is NOT English or German.
**E3**	The publication is NOT an article in a peer-reviewed Journal (e.g. dissertation abstract, conference abstract).
**E4**	The main aim of the study is NOT to test psychometric properties of an instrument.
**E5**	The measured construct is NOT information needs.
**E6**	The instrument does NOT measure information needs of an adult patient (e.g. of relatives, physicians, children, computer user etc.).
**E7**	Information needs is only a subscale of the instrument and results are NOT reported for this subscale explicitly (e.g. within communication needs).

### Study selection

To facilitate the study selection process, we imported the search results into the reference management software EndNote and removed duplicates. First, two reviewers (WF, EC; update: NK, EC) independently conducted a title and abstract screening to exclude clearly irrelevant records. All articles that were found possibly relevant by one researcher were included. Second, the remaining full texts were independently assessed for eligibility by two reviewers (WF, EC; update: NK, EC) using the inclusion and exclusion criteria. Full text disagreements between reviewers were resolved by discussion; where necessary, a third reviewer (IS, update: JZ) was consulted.

### Data extraction and quality assessments

Data extraction was conducted by using a data extraction sheet, which was already used in other systematic reviews of measures conducted by the research team [15–18]. The data extraction sheet contained both descriptive data and data to assess the quality of the included studies. The process of assessing the quality consisted of two distinct steps. First, the methodological quality of the studies was assessed by applying the COnsensus-based Standards for the selection of health Measurement INstruments (COSMIN) checklist with a 4-point scale [19]. Second, the quality of the psychometric properties of the identified rating scales was assessed using the quality criteria developed by Terwee et al. [20]. Both rating instruments are described below. To reduce any bias that may occur through single reviewer assessment, two randomly selected studies were extracted and assessed independently by two (EC and IS). The remaining studies were extracted and assessed by one reviewer only (EC).

#### Assessment of methodological quality

The COSMIN criteria were developed in an international Delphi study, which aimed at developing consensus on definitions and assessments of measurement properties [21]. Nine boxes of the COSMIN checklist [19] refer to methodological standards for studies on measurement properties: internal consistency, reliability, measurement error, content validity, structural validity, hypotheses testing, cross-cultural validity, criterion validity, responsiveness. Each of the boxes consists of several items on design requirements and statistical analyses. The items can be scored on a 4-point rating scale as poor (0), fair (+), good (++) or excellent (+++). To determine the overall score of each box, the lowest score of any item within the box has to be taken (so called “worst score counts” method). For studies based on item response theory (IRT) four items on IRT have to be scored first and the “worst score counts” method has to be applied on each box in combination with those four items.

#### Assessment of psychometric quality

For the evaluation of the quality of psychometric properties of identified measures we applied the criteria developed by Terwee et al. [20]. These criteria refer to content validity, internal consistency, criterion validity, construct validity, reproducibility (agreement and reliability), responsiveness, floor and ceiling effects, and interpretability. Each of these psychometric properties can be rated by one item as either positive (+), intermediate (?), negative (-), or no information available (0).

#### Data analysis and synthesis of results

Main characteristics of the included studies and measurements as well as the assessment of the methodological quality and the psychometric quality were combined in a narrative summary. An overview of the results of the quality ratings is also displayed in two tables.

## Results

### Literature search and study selection

The first original electronic search yielded 6711 records and the secondary searches identified another 55 records. After removal of duplicates, 4889 records remained. Through title and abstract screening 4842 records were excluded and the remaining 47 full-texts were assessed for eligibility. A total of 29 full-texts were excluded according to the predefined criteria (see Table 2) and the remaining 18 full articles were included in the study. In the update of the electronic search 1690 references were found and after duplicate removal 1208 title and abstracts were screened. One article was included from the primary search, and the secondary search added another five articles.

**Fig 1:**
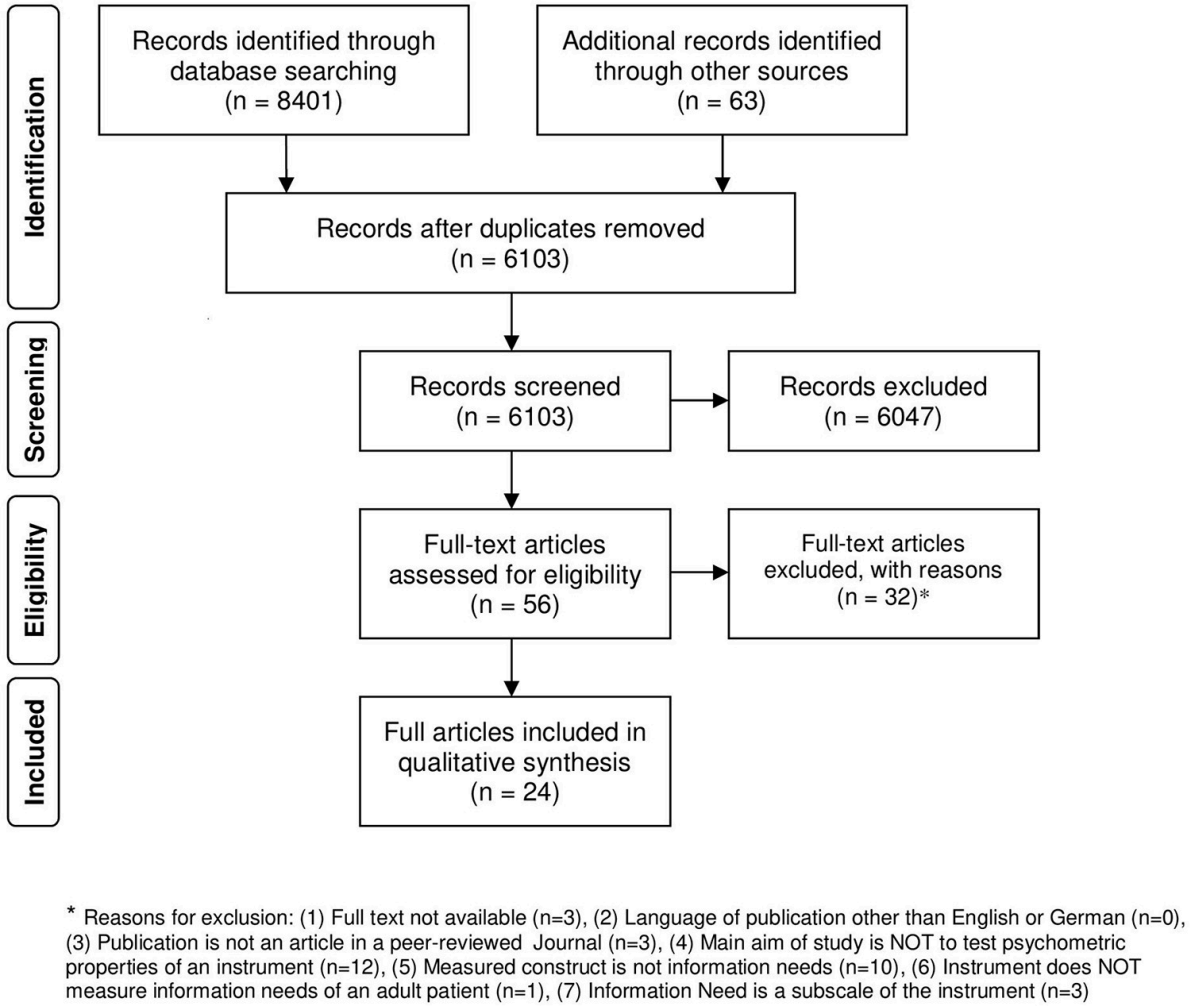
Flow diagram of study selection.

This resulted in 24 included studies overall from the original search and the update. The flow diagram of study selection is displayed in Fig 1. An overview on the reasons for exclusion of full texts is given in the S2 Appendix.

### Description of included studies and measures

The 24 included studies were conducted in a range of countries and continents, with most studies coming from Europe [22–34]. Sample sizes ranged from N = 28 [35] to N = 3015 patients [30] and the studies were conducted in a range of diverse settings (e.g. hospitals, outpatient clinics and self-help groups). Descriptive data of the included studies are shown in Table 3.

**Table 3:** Descriptive data of the included studies.

*First author*, *Year*	*Country*	*Focus*	*Setting*	*Study Sample*
Alamanou, 2016	Greece	cancer	in- and outpatients from public hospital in Athens	n = 109, 56% male, age: median 65.5 years, SD 11.9
Bubela, 1990	Not reported	hospital discharge	metropolitan hospital	n = 301, 50% male, age: median 53.8 (range 18–80)
Czar, 1997	USA	angina pectoris/ myocardial infarction	university-affiliated medical center and clinic	n = 28, 100% male, age: M = 61 years (range 31–80)
Dale, 2004	UK	prostate cancer	urology outpatient clinics	n = 96, 100% male, age: M = 73 years (range 57–93)
Dall'Armi, 2013	Australia	head and neck cancer	regionally defined cancer services	n = 79, 80% male, age: M = 62.7 years (SD 11.9)
Degner, 1998	Canada, UK	cancer	not reported	n = 150 (UK), n = 1012 (Canada)
Fitch, 2011	Canada	cancer	outpatients	n = 540, 53% female, age: M = 60.9 years (SD 14.5)
Galdeano, 2014	Portugal	coronary artery disease	coronary intensive care unit and cardiothoracic surgery unit of a hospital	n = 200, 76% male, age: M = 65 years (SD 11.8)
Galloway, 1997	Canada	breast cancer	urban hospital and outpatient cancer clinic	n = 70, 100% female, age: M = 53.9 (SD 12.6, range 21–91)
Ghisi, 2013	Canada	cardiac rehabilitation	participants of CR programs	n = 203, 24.1% female, age: M = 64 years (SD 11.8)
Ghisi, 2014	Brazil	cardiac rehabilitation	participants of CR programs	n = 300, 66% male, age: M = 61.3 years (SD 2.1, range 35–85)
Halkett, 2007	Australia	radiotherapy for breast cancer	media and radiation oncologist at a hospital	n = 30, 100% female, age: median 55.2 years (SD 9.6, range 33–74)
Hardware, 2004	UK	arthritis	hospital rheumatology outpatient clinic	n = 97, 63.2% female, age: <60 n = 36/ 60+ n = 39
Meesters, 2009	Netherlands	rheumatic diseases	rheumatology outpatient clinic in medical university center	n = 165, 88.55% female, age: median 68 (range 55–77)
Mesters, 2001	Netherlands	cancer	university hospital	n = 133, 84% male
Ndosi, 2011	Austria, Finland, Spain, Norway, Netherlands, Portugal, Sweden	rheumatic diseases	rheumatology outpatient clinics, day units, inpatient wards, databases, rehabilitation centers and/or from the community	n = 3015, 66.2% female, age: median 52.6, SD 13.1
Nokes, 1997	USA	HIV	four tertiary care facilities	n = 363, 88% female, age: brackets: 21–29 n = 32/ 20–29 n = 135/ 40–49 n = 147/ 50–59 n = 28/ 60+ n = 20
O'Connor, 2010	Northern Ireland	prostate cancer	former hospital patients	n = 40, 40% female, age: M = 66.6 years (SD 11.5, range 44–86)
Ormandy, 2013	UK	chronic kidney disease	dialysis clinical network	n = 89, 59,6% male, age: M = 56.7 years (median = 59, range 25–83)
Piredda, 2008	Italy	chemotherapy	university hospital	n = 108, 64% male, age: median 60 years (range 28–80)
Templeton, 2001	Northern Ireland	prostate cancer	urology units	n = 90
Yi et al, 2007	Korea	breast cancer	self-support groups	n = 164, 100% female, age: M = 49 years (range 30–73)
Yu, 2010	China	heart failure	university-affiliated general hospitals	n = 247, 64% male, age: 64% > 60 years
Zeguers, 2010	Netherlands	radiotherapy	outpatient radiotherapy department	n = 154, 60% male, age: median 63 years (range 19–88)

M = mean, SD = standard deviation.

The 24 included studies report on the psychometric properties for 21 distinct measures. Three measures have been psychometrically tested in two studies each. First, this was the Information Needs in Cardiac Rehabilitation measure [36, 37]. Second, was a measure on informational needs of men with prostate cancer by Templeton et al. [33] with further testing by O’Connor et al. [31]. Last is the Educational Needs Assessment Tool (ENAT) which had been tested in seven European countries [30] and had been partly reported for the Dutch version in an earlier publication [28]. While twelve measures focus on cancer or cancer treatment information [24–26, 29, 32–34, 38–42], four assess patient information needs related to heart disease [22, 35, 36, 43] and one instrument each on information needs regarding chronic kidney disease [23], arthritis [27], rheumatic diseases [30], HIV [44] and at discharge from hospital in general [9]. All measures are multi-dimensional and range from nine items [26] to 55 items [36].

Descriptive data of the included measures are shown in Table 4.

**Table 4:** Characteristics of information needs measures.

*Initial study*	*Measure*	*Focus*	*Language*	*Domains (D) and Items (I)*	*Response scale*
Alamanou, 2016	Greek Cassileth's Information Styles Questionnaire	cancer	Greek	50 items in 2 domains (disease and treatment, psychological)	
Bubela, 1990	Patient Learning Needs Scale*	discharge from hospital, disease-generic	English	75 items in 7 domains (medications, living activities, skin care, community and follow-up, feelings related to condition, treatments and complications, enhancing quality of life)	6-point-Likert
Czar, 1997	Everything You Ever Wanted To Know About Heart Disease	angina pectoris or myocardial infarction	English	38 items in 10 domains (cardiac anatomy and physiology, food restrictions, exercise, recognizing symptoms, sex, medications, smoking, work, stress, general concerns)	5-point Likert
Dale, 2004	Information Needs of Men With Prostate Cancer	prostate cancer	English	20 items in 4 domains (basics of prostate cancer care, disease management, psychosocial & physical wellbeing, self-help)	4-point Likert
Dall'Armi, 2013	Head and Neck Information Needs Questionnaire (HaNiQ)	head and neck cancer	English	33 items in 5 domains (disease profile, treatment, side effects, psychosocial, survivorship)	4-point Likert
Degner, 1998	Information Needs in Cancer Care*	cancer	English	9 items in 9 domains (stage of disease, likelihood of cure, effect of treatment on social activity, effect of disease on family and close friends, self-care needs, effect of treatment on usual sexual activity, types of treatment available, risk of a family member developing the disease, side effects of treatment)	Thurstone
Fitch, 2011	Cancer Patient Information Importance Scale	cancer	English	12 items in 2 domain (importance of information, satisfaction of information, for the present paper only the first scale had been reviewed)	5-point Likert
Galdeano, 2014	Cardiac Patients Learning Needs Inventory (CPLNI)—Portuguese Version	coronary artery disease	Portuguese	43 items in 8 domains (introduction to the critical care unit, anatomy and physiology, psychological factors, risk factors, medication information, diet information, physical activity, other pertinent information)	5-point Likert
Galloway, 1997	Toronto Informational Needs questionnaire-Breast Cancer (TINQ-BC)	breast cancer	English	51 items in 6 domains (diagnosis, investigative tests, treatments, physical, psychological, family)	5-point Likert
Ghisi, 2013	Information Needs in Cardiac Rehabilitation (INCR)	cardiac rehabilitation	English, Portuguese	55 items in 10 domains (the heart, nutrition, exercise/ physical activity, medication, work/ vocational/ social, stress/ psychological factors, general/ social concerns, emergency/ safety, diagnosis, treatment, risk factors)	5-point Likert
Halkett, 2007	RT Information Needs Scale	radiation therapy for breast cancer	English	22 items in 4 domains (initial information about radiation therapy, information relating to planning treatment, information relating to first day of treatment, effect treatment will have on day to day living during treatment)	Not reported
Hardware, 2004	Educational Needs in Patients With Arthritis*	arthritis	English	39 items in 7 domains (managing pain, movement, feelings, arthritis, treatment from health professionals, self-help measures, support from others)	5-point Likert
Mesters, 2001	Patient Information Need Questionnaire (PINQ)	cancer	Dutch	17 items in 2 domains (disease-oriented, action-oriented)	4-point Likert
Ndosi, 2011	Educational Needs Assessment Tool (ENAT)	rheumatic diseases	English, Finnish, Dutch, Norwegian, Portuguese, Spanish, Swedish	39 items in 7 domains (managing pain, movement, feelings, disease process, treatments, self-help measures, support systems)	5-point Likert
Nokes, 1997	HENAT (HIV Educational Needs Assessment Tool)	HIV	English	34 items in 6 domains (treatments, entitlements, relationships, preventing infections, social support, working subscales)	5-point Likert
Ormandy, 2013	Patients' Preferences and Priorities for Information in Chronic Kidney Disease*	chronic kidney disease	English	45 items in 12 domains (domain names shortened by author: chronic kidney disease and progression, physical effects of renal replacement therapy (RRT), RRT in general, practical aspects of RRT, complication and side effects of RRT, medication and side effects, family and lifestyle, work and finances, diet and fluid restrictions, tests and blood results, psychological issues, other patient experiences	Thurstone
Piredda, 2008	Learning Needs in Oncology Patients Receiving Chemotherapy*	cancer and chemotherapy	Italian	11 items, no subscales	5-point Likert
Templeton, 2001	Informational Needs of Men With Prostate Cancer*	prostate cancer	English	29 items in 5 domains (disease, physical, treatment, psychosocial, investigative tests)	5-point Likert
Yi et al, 2007	Korean Informational Needs Questionnaire of Breast Cancer	breast cancer	Korean	52 items in 5 domains (disease, treatment, investigative tests, psychosocial, physical needs)	5-point Likert
Yu, 2010	Heart Failure Learning Needs Inventory—Patient Section (HFNLI)	heart failure	Chinese	47 items in 9 domains (general HF information, psychologic factors, risk factors, medications, diet, activity, prognosis, signs, symptoms)	5-point Likert
Zeguers, 2010	Information Preferences Of Radio-therapy Patients Questionnaire (IPRP)	radiotherapy	Dutch	34 items in 6 domains (disease, prognosis, treatment, radiotherapy procedures, radiotherapy side effects, psychosocial information)	5-point Likert

* For the indicated instruments no name had been reported, thus we created a name based on the article. n/r = not reported.

### Methodological quality of included studies

The assessment of the methodological quality of the included studies by applying the COSMIN checklist is displayed in Table 5. Included studies reported a mean of 2.8 out of the nine COSMIN criteria. For two studies only content validity could be rated as their measurement used a differential scale (Thurstone scale) [23, 26]. Only one study used a measure based on IRT [30] and scored “good” on the four IRT-Items.

**Table 5:** Assessment of the methodological quality with COSMIN criteria.

*First author*, *Year*	*Focus*	*Psychometric properties*									
		IRT	A	B	C	D	E	F	G	H	I
Alamanou, 2016	cancer		0				0				
Bubela, 1990	hospital discharge		+			+	0				
Czar, 1997	angina pectoris/myocardial infarction		0								
Dale, 2004	prostate cancer		0			+	0				
Dall'Armi, 2013	head and neck Cancer		0			0	0				
Degner, 1998	cancer					++					
Fitch, 2011	cancer		0			0					
Galdeano, 2014	coronary artery disease		0	0					++		
Galloway, 1997	breast cancer		0			0					
Ghisi, 2013	cardiac rehabilitation		0	0		0					
Ghisi, 2014	cardiac rehabilitation		+	+					++		
Halkett, 2007	radiotherapy for breast cancer		0	0		+					
Hardware, 2004	arthritis			+		0					
Meesters, 2009	rheumatic diseases		0						+		
Mesters, 2001	cancer		+			0	+	0			
Ndosi, 2011	rheumatic diseases	++	+					+	+		
Nokes, 1997	HIV		+	+		0	+	+			
O'Connor, 2010	prostate cancer		0			+					
Ormandy, 2013	chronic kidney disease					+					
Piredda, 2008	chemotherapy		0			0					
Templeton, 2001	prostate cancer		0			0	0				
Yi, 2007	breast cancer		0			0	0		+++		
Yu, 2010	heart failure		+	+		0	+		0		
Zeguers, 2010	radiotherapy		0					0			

COSMIN psychometric property boxes: IRT = item response theory, A = internal consistency, B = reliability, C = measurement error, D = content validity, E = structural validity, F = hypotheses testing, G = cross-cultural validity, H = criterion validity, I = responsiveness. 4-point scale rating: +++ = excellent, ++ = good, + = fair, 0 = poor, empty space = COSMIN rating not applicable. For exact information regarding the definitions of psychometric properties and 4-point scale rating see COSMIN website (www.cosmin.nl).

Internal consistency was calculated in 21 out of 24 included studies. Only six of them received a “fair” score [9, 29, 30, 37, 43, 44], while 15 scored “poor” [22, 24, 25, 28, 31–36, 38–42]. The second most commonly assessed property was content validity, which was assessed in 17 studies. Eleven of those studies received a “poor” [27, 29, 32, 33, 36, 38–40, 42–44], five a “fair” [9, 23–25, 31, 41] and one a “good” score [26] for content validity. Structural validity was assessed in nine studies, with six scoring “poor” [9, 24, 25, 33, 38, 42] and three scoring “fair” [29, 43, 44]. Scores for reliability were “poor” in three studies [22, 36, 41] and “fair” for four studies [27, 37, 43, 44]. Of the six studies evaluating cross-cultural validity, only one was rated as “poor” [43], while two scored “fair” [28, 30], two received a “good” [22, 37] and one an “excellent” [42] score. Hypothesis testing was done in four studies, of which two performed “poorly” [29, 34] and two were rated as “good” [30, 44]. No studies assessed measurement error, criterion validity and responsiveness.

### Quality of psychometric properties

The assessment of the psychometric quality of the identified measures by applying the Terwee criteria [20] is displayed in Table 6. Most included studies reported three or four out of the nine criteria. Again, two studies were only rated for content validity due to their differential scale [23, 26]. In addition, the study by Ndosi et al. [30] had not been rated as the Terwee criteria do not apply to IRT.

**Table 6:** Quality rating of psychometric properties with Terwee criteria.

*First author*, *Year*	*Focus*	*Psychometric properties* *For exact information regarding the definitions of psychometric properties see* [45].								
		*Content validity*	*Internal consistency*	*Criterion validity*	*Construct validity*	*Reproducibility Agreement*	*Reproducibility Reliability*	*Responsiveness*	*Floor & ceiling effects*	*Interpretability*
Alamanou, 2016	cancer	0	?	0	0	0	0	0	0	?
Bubela, 1990	hospital discharge	+	?	0	0	0	0	0	0	0
Czar, 1997	angina pectoris/myocardial infarction	0	?	0	0	0	0	0	0	?
Dale, 2004	prostate cancer	+	?	0	0	0	0	0	0	?
Dall'Armi, 2013	Head and Neck Cancer	-	?	0	0	0	0	0	0	?
Degner, 1998^a^	cancer	+								
Fitch, 2011	cancer	?	?	0	0	0	0	0	0	?
Galdeano, 2014	coronary artery disease	?	?	0	0	0	?	0	0	?
Galloway, 1997	breast cancer	+	?	0	0	0	0	0	?	?
Ghisi, 2013	cardiac rehabilitation	?	?	0	0	0	?	0	0	?
Ghisi, 2014	cardiac rehabilitation	0	?	0	0	0	+	0	0	?
Halkett, 2007	radiotherapy for breast cancer	+	?	0	0	0	-	0	0	?
Hardware, 2004	arthritis	?	0	0	0	0	+	0	0	?
Meesters, 2009	rheumatic diseases	0	?	0	0	0	0	0	0	?
Mesters, 2001	cancer	0	+	0	+	0	0	0	0	?
Ndosi, 2011^b^	rheumatic diseases									
Nokes, 1997	HIV	?	+	0	?	0	0	0	0	0
O'Connor, 2010	prostate cancer	?	?	0	0	0	0	0	0	?
Ormandy, 2013^a^	chronic kidney disease	+								
Piredda, 2008	chemotherapy	?	?	0	0	0	0	0	0	?
Templeton, 2001	prostate cancer	?	?	0	0	0	0	0	0	?
Yi et al, 2007	breast cancer	?	?	0	0	0	0	0	0	?
Yu, 2010	heart failure	?	?	0	0	0	+	0	0	?
Zeguers, 2010	radiotherapy	0	?	0	?	0	0	0	0	?

Rating: + = positive, ? = intermediate, − = negative, 0 = no information available.

^a^ Only content validity had been rated as they used a differential scale.

^b^ No rating as the Terwee criteria do not apply to IRT, which had been used here.

All in all, the psychometric properties of the identified measures were mainly rated as “intermediate” with some “positive” ratings. The most commonly assessed criteria was internal consistency in 20 studies with two “positive” results [29, 44] and the rest being rated “intermediate”. This was followed by content validity which had been assessed in 17 studies with six “positive” [9, 23, 25, 26, 40, 41] and one “negative” [38] rating while the remaining studies received “intermediate” results.

## Discussion

The aim of this review was to systematically identify and assess psychometric studies on measures of patient information needs, to assess the methodological quality of these studies and to investigate the quality of the psychometric properties of the identified questionnaires. To our knowledge, this is the first systematic review that systematically appraises and summarizes the existing evidence on measures on patient information needs. Our systematic literature search revealed 24 studies assessing 21 measures on patient information needs.

The methodological quality of the included studies with regard to quality of design, methods and reporting was predominantly rated from “poor” to “fair”. Reliability was evaluated in only seven measures, and if reliability has been assessed, the quality was judged as “fair” at best. The poor reliability assessments may be partly due to the “worst case counts” as a conservative method of the COSMIN checklist, i.e. the lowest score of any item in a box is considered the overall score of that box. Consequently, if a study is of good methodological quality but does not provide this information in the study (rated “fair”), the highest score in that box can only be “fair”. This emphasizes the importance of further improvements of the reporting of studies on measurement properties in the literature.

Although reported more often, the evidence for content validity was weak and rated as “poor” in eleven measures. Only the measure used in the study of Degner et al. [26] was rated as “good”. Moreover, the evidence for the structural validity of the included measures was also weak. In 15 measures, structural validity was not analyzed, the remaining 6 tools were rated “fair” at best. Another weakness was the lack of reported hypotheses testing, criterion validity and whether the tools are sensitive to change. Although there was only limited information on cross-cultural validity (six measures) the ratings predominantly ranged between “fair” and “good”. Remarkably, only one study scored "excellent" on at least one psychometric property when using the COSMIN-checklist.

Overall, the rated standards for design requirements and preferred statistical methods (mean of 2.8) showed mostly “poor” quality. The best results were shown for the measure of Ndosi et al. [30] for rheumatic diseases, which is based on the item response theory and had been tested in a large population in seven European countries. A second study with satisfactory quality was the one conducted by Ghisi et al. [37] on an instrument for the use in cardiac rehabilitation.

Similar results were shown when rating the quality of psychometric properties by applying the Terwee criteria. For many of the psychometric properties, there was only limited information available. Exceptions could be seen for the criteria "content validity", on which ratings mostly ranged from "intermediate" to "positive", and the criteria "internal consistency" and "interpretability" on which ratings were predominantly "intermediate". However, these limited quality ratings on the Terwee criteria partly reflect the low ratings on the COSMIN-checklist. It is likely that some of the measures received a low rating due to incomplete reporting within these studies. The questionnaire used in the study by Mesters et al. [29] for cancer patients was judged as psychometrically most promising.

From our findings, we clearly see a need for more evaluation studies on existing measures, focusing on psychometric properties that have not been tested satisfactorily (e.g. reliability, content validity) or hardly or not tested at all for most measures (e.g. structural validity, responsiveness). Moreover, more rigorous study designs and better reporting, including adherence to the COSMIN-checklist, are needed. The results of this review show that there is also a need for the development of new measures with good psychometric properties as there are no measures for a range of frequent chronic diseases (e.g. diabetes, COPD and asthma, depression, chronic back pain). Until today, there is lack of measures on information needs for many indications. Therefore, in clinical practice it is also important to concentrate on other forms of assessing patient information needs. Timmins [8] suggested the provision of an open-style interview, in which the clinician systematically asks patients and their caregivers about their information needs on the respective disease and its treatment. Although this is a time-consuming procedure, the result might be more comprehensive than other interventions (e.g. checklists). Another option is the training of patients to enable them to ask their questions. Yet a Cochrane Review showed that interventions (e.g. question prompt sheets) used before consultations for helping patients to address their information needs showed limited benefits [5].

### Strengths and limitations of the study

Strengths of this review are the following. First, we conducted a detailed and recently updated electronic search. Second, two reviewers independently assessed all abstracts and full texts. Third, we used both the COSMIN checklist and the quality criteria for good psychometric properties developed by Terwee et al. [20] for quality assessment.

Limitations of this review are, first, that we excluded measures that assessed information needs as a subscale of a broader measure for reasons of feasibility. Second as data extraction is very time consuming, not all data had been extracted by two researchers. Yet to ensure that the data extraction sheet is clear and yields reliable results, two studies had been independently extracted by two researchers (EC and IS) in the beginning. Furthermore if there had been any doubt on how to extract certain aspects of a study the first researcher (EC) had consulted the second researcher (IS). Finally, we limited our search to two databases and might have missed relevant articles. However, our search strategy was very sensitive and we also added a secondary search to detect studies that were not found by the electronic search.

## Conclusion

This is the first systematic review of studies on measures that assess information needs. Due to a comprehensive evaluation of the methodical quality of the included studies and the psychometric properties of the measures the results of this review can help researchers and clinicians to choose the right measure for their research purpose or clinical consultation. Moreover, this review shows a strong need for further evaluation and testing of current measures in this field, including the application of standards like the COSMIN checklist. There is also a need for the development of new measures, as our results show that only for a few conditions specific measures on information needs are available yet.

## Supporting information

S1 Appendix PRISMA checklist. (DOC)

S2 Appendix Reasons for exclusion. (DOCX)
