# Supplementary material for: Assessment of patient information needs: A systematic review of measures
Source: PLoS One. 2019 Jan 31;14(1):e0209165. doi: 10.1371/journal.pone.0209165 (PMC6354974; doi:10.1371/journal.pone.0209165)
Supplement: S2 Appendix — (DOCX) [file pone.0209165.s002.docx]

*Appendix S2: Reasons for exclusion in fulltext screening*

| Exclusion criteria | Reference |
| --- | --- |
| E5 Measured construct is NOT information needs | Arraras JI, Wright S, Greimel E, Holzner B, Kuljanic-Vlasic K, Velikova G, et al. Development of a questionnaire to evaluate the information needs of cancer patients: the EORTC questionnaire. Patient Educ Couns. 2004;54(2):235-41. Epub 2004/08/04. |
| E5 Measured construct is NOT information needs | Astrom K, Carlsson J, Bates I, Webb DG, Duggan C, Sanghani P, et al. Desire for information about drugs. A multi-method study in general medical inpatients. Pharmacy world & science : PWS. 2000;22(4):159-64. Epub 2000/12/05. |
| E4 Main aim was NOT psychometric testing | Buzaglo JS, Millard JL, Ridgway CG, Ross EA, Antaramian SP, Miller SM, et al. An internet method to assess cancer patient information needs and enhance doctor-patient communication: A pilot study. Journal of Cancer Education. 2007;22(4):233-40. |
| E4 Main aim was NOT psychometric testing | Carlson ML, Ivnik MA, Dierkhising RA, O'Byrne MM, Vickers KS. A learning needs assessment of patients with COPD. Medsurg nursing : official journal of the Academy of Medical-Surgical Nurses. 2006;15(4):204-12. Epub 2006/09/27. |
| E4 Main aim was NOT psychometric testing | Chernyak N, Stephan A, Bachle C, Genz J, Julich F, Icks A. Assessment of information needs in diabetes: Development and evaluation of a questionnaire. Primary care diabetes. 2016. Epub 2016/01/19. |
| E6 NOT for adult patients | Cooper H, Spencer J, Lancaster GA, Titman A, Johnson M, Wheeler SL, et al. Development and psychometric testing of the online Adolescent Diabetes Needs Assessment Tool (ADNAT). Journal of Advanced Nursing. 2014;70(2):454-68. |
| E4 Main aim was NOT psychometric testing | Czar ML, Engler MM. Perceived learning needs of patients with coronary artery disease using a questionnaire assessment tool. Heart & lung : the journal of critical care. 1997;26(2):109-17. Epub 1997/03/01. |
| E4 Main aim was NOT psychometric testing | Degner LF, Kristjanson LJ, Bowman D, Sloan JA, Carriere KC, O'Neil J, et al. Information needs and decisional preferences in women with breast cancer. Jama. 1997;277(18):1485-92. Epub 1997/05/14. |
| E5 Measured construct is NOT information needs | Droege M, Leonard ST, Duggan CA. Cross-cultural validation of the desire for information about prescribed medicines scale. The International journal of pharmacy practice. 2010;18(1):37-42. Epub 2010/04/22. |
| E5 Measured construct is NOT information needs | Duggan C, Bates I, Sturman S, Andersson E, Astrom K, Carlsson J. Validation of a "desire for information" scale. International Journal of Pharmacy Practice. 2002;10(1):31-7. |
| E4 Main aim was NOT psychometric testing | Fitch MI, McAndrew A, Harth T. Measuring trends in performance across time: providing information to cancer patients. Canadian oncology nursing journal = Revue canadienne de nursing oncologique. 2013;23(4):247-61. |
| E1 Fulltext not available | Galloway S. Measuring information needs of women with breast cancer. Canadian Oncology Nursing Journal. 1994;4(29). |
| E5 Measured construct is NOT information needs | Garvin BJ, Kim CJ. Measurement of preference for information in U.S. and Korean cardiac catheterization patients. Research in nursing & health. 2000;23(4):310-8. |
| E1 Fulltext not available | Gerard PS, Peterson LM. Learning needs of cardiac patients. Cardio-vascular nursing. 1984;20(2):7-11. Epub 1984/03/01. |
| E4 Main aim was NOT psychometric testing | Hagenhoff BD, Feutz C, Conn VS, Sagehorn KK, Moranville-Hunziker M. Patient education needs as reported by congestive heart failure patients and their nurses. Journal of advanced nursing. 1994;19(4):685-90. Epub 1994/04/01. |
| E5 Measured construct is NOT information needs | Hahn U, Hechler T, Witt U, Krummenauer F. Konzeption und Inhaltsvalidierung eines Fragebogens zu Informationsbedürfnissen bezüglich Dienstleistungen und Ansprechpartnern von sehbehinderten Menschen (FIDAS) Conception and Content Validation of a Questionnaire Relating to the Potential Need for Information of Visually Impaired Persons with Regard to Services and Contact Persons. Klin Monatsbl Augenheilkd. 2015;232(12):1402-9. |
| E1 Fulltext not available | Hughes M. An instrument to assist nurses identify patients’ self perceived informational needs post myocardial infarction. All Ireland Journal of Nursing and Midwifery. 2000;1(1):13-7. |
| E5 Measured construct is NOT information needs | Hyland ME, Jones RC, Hanney KE. The Lung Information Needs Questionnaire: Development, preliminary validation and findings. Respir Med. 2006;100(10):1807-16. Epub 2006/03/10. |
| E7 information needs is only a subscale | Lile JB, Buhmann J, Roders S. Development of a learning needs assessment tool for patients with congestive heart failure. Home Health Care Management & Practice. 1999;11(6):11-25. |
| E7 information needs is only a subscale | Loerbroks A, Leucht V, Keuneke S, Apfelbacher CJ, Sheikh A, Angerer P. Patients' needs in asthma treatment: development and initial validation of the NEAT questionnaire. The Journal of asthma : official journal of the Association for the Care of Asthma. 2016;53(4):427-37. Epub 2016/01/21. |
| E5 Measured construct is NOT information needs | Maibach EW, Weber D, Massett H, Hancock GR, Price S. Understanding consumers' health information preferences development and validation of a brief screening instrument. Journal of Health Communication. 2006;11(8):717-36. |
| E4 Main aim was NOT psychometric testing | Mooney J, Spalding N, Poland F, Grayson P, Leduc R, McAlear CA, et al. The informational needs of patients with ANCA-associated vasculitis-development of an informational needs questionnaire. Rheumatology. 2014;53(8):1414-21 |
| E5 Measured construct is NOT information needs | Murtagh FE, Thorns A. Evaluation and ethical review of a tool to explore patient preferences for information and involvement in decision making. Journal of medical ethics. 2006;32(6):311-5. Epub 2006/05/30. |
| E3 NOT an article in peer-reviewed journal | Passalacqua S, Mozzetta A, Di Pietro C, Marchetti P, Tabolli S. Information needs: A study about oncological patients in the Italian context. Value in Health. 2009;12 (7):A283. |
| E4 Main aim was NOT psychometric testing | Sauro A, Greco A, Lo Greco P, Scalzitti F, Sirignano AR, Sortino D, et al. The COPD Italian Lung Information Needs Questionnaire (LINQ): development, preliminary validation, and findings. Eur J Gen Pract. 2008;14(2):65-7. Epub 2008/09/30. |
| E4 Main aim was NOT psychometric testing | Scott JT, Thompson DR. Assessing the information needs of post-myocardial infarction patients: a systematic review. Patient education and counseling. 2003;50(2):167-77. Epub 2003/06/05. |
| E4 Main aim was NOT psychometric testing | Spyropoulos V, Ampleman S, Miousse C, Purden M. Cardiac surgery discharge questionnaires: meeting information needs of patients and families. Canadian journal of cardiovascular nursing = Journal canadien en soins infirmiers cardio-vasculaires. 2011;21(1):13-9. |
| E7 information needs is only a subscale | Tamburini M, Gangeri L, Brunelli C, Beltrami E, Boeri P, Borreani C, et al. Assessment of hospitalised cancer patients' needs by the Needs Evaluation Questionnaire. Annals of oncology : official journal of the European Society for Medical Oncology / ESMO. 2000;11(1):31-7. Epub 2000/02/26. |
| E5 Measured construct is NOT information needs | Ter Hoeven CL, Zandbelt LC, Fransen S, De Haes H, Oort F, Geijsen D, et al. Measuring cancer patients' reasons for their information preference: Construction of the Considerations Concerning Cancer Information (CCCI) questionnaire. Psycho-Oncology. 2011;20(11):1228-35. |
| E3 NOT an article in peer-reviewed journal | Thabane M, Chauhan U, Wolfe MA, Harper T, Akhtar-Danesh N, Simunovic M, et al. Information needs of digestive disease patients: The patient education needs survey (PENS). Gastroenterology. 2009;1):A638. |
| E3 NOT an article in peer-reviewed journal | Uysal H, Enc N. Adaptation of the cardiac patients' learning needs inventory; patient questionnaire to in Turkish, Kalp hastalarinin etitim gereksinimlerinin incelenditi hasta soru formunun Turkceye uyarlanmasi. [Turkish, English]. Turk Kardiyoloji Dernegi Arsivi. 2011;39:258. |
| E4 Main aim was NOT psychometric testing | Zwaenepoel L, Hoorens V, Peuskens J, Laekeman G, Group VZ-PR. The "extent of information desired"-scale in psychiatric in-patients: a behavioural approach. Patient Educ Couns. 2006;62(1):72-8. Epub 2006/02/25. |
